# Outbreak of *Salmonella* Typhimurium ST19 linked to passerine birds and cats in Norway, March to July 2024

**DOI:** 10.1007/s10096-026-05421-8

**Published:** 2026-02-05

**Authors:** Hilde Marie Lund, Lin T. Brandal, Liz Ertzeid Ødeskaug, Heidi Lange, Polina Katsiouleri, Gro S. Johannessen, Bjarne Bergsjø, Åsne Sangolt, Rikard Dryselius, Nadja Karamehmedovic, Henry Kuronen, Anni Vainio, Ruska Rimhanen-Finne, Umaer Naseer

**Affiliations:** 1 Department of Infection Control and Preparedness, Norwegian Institute of Public Health, Oslo, Norway; 2 Department of Bacteriology, Norwegian Institute of Public Health, Oslo, Norway; 3 Norwegian Veterinary Institute, Ås, Norway; 4 Regulations and Control Department, Norwegian Food Safety Authority, Bergen, Norway; 5 Department of Communicable Disease Control and Health Protection, Public Health Agency of Sweden, Solna, Sweden; 6 Department of Microbiology, Public Health Agency of Sweden, Solna, Sweden; 7 Laboratory and Research Division, Finnish Food Authority, Kuopio, Finland; 8 Department of Public Health, National Institute for Health and Welfare (THL), Helsinki, Finland

**Keywords:** *Salmonella* outbreak, Passerine birds, Cats, Whole genome sequencing, Norway, Nordic countries

## Abstract

**Purpose:**

In June 2024, a genomic cluster of seven *Salmonella* Typhimurium - sequence type 19, and cluster type 21092 - was detected in Norway, triggering a national outbreak investigation.

**Methods:**

Information about new cases was collected from the Norwegian Surveillance System for Communicable Diseases and the database at the National Reference Laboratory for Enteropathogenic Bacteria (NRL) at the Norwegian Institute of Public Health (NIPH). Microbiological analyses were conducted by NRL at NIPH for human samples and at the Norwegian Veterinary Institute for animal samples. Epidemiological data was collected through interviews. International notification was sent via EpiPulse.

**Results:**

Eleven cases in total, sampled between March 6 and July 11, 2024, were identified across Norway. The median age of affected individuals was three years. Notably, 73% of the cases reported prior contact with cats or passerine birds. The outbreak strain was also detected in a faecal sample from a cat belonging to one of the affected households, suggesting an animal source. Concurrently, Finland and Sweden reported five and six cases, respectively, involving the same outbreak strain. Several of these individuals also reported contact with cats or birds.

**Conclusions:**

Passerine birds are a well-documented reservoir for *S.* Typhimurium in the Nordic region, often leading to transmission to both cats and humans. This outbreak highlights the role of animal exposure in the spread of *S.* Typhimurium and emphasize the need for timely, targeted public health communication on infection prevention measures.

**Supplementary Information:**

The online version contains supplementary material available at 10.1007/s10096-026-05421-8.

## Introduction

Salmonellosis is a zoonotic disease caused by non-typhoidal *Salmonella*. In 2023, salmonellosis was the second most frequently reported foodborne zoonosis in the European Union/European Economic Area (EU/EEA) following campylobacteriosis, with a notification rate of 18.0 per 100 000 population [1]. The reservoir for *Salmonella* is diverse, including humans and most warm- and cold-blooded animals, with over 2 500 different serovars described [2].

*Salmonella* spp. typically causes self-limiting gastrointestinal illness, but infections can also lead to more severe clinical outcomes. The incubation period usually ranges from 6 to 72 h [3].

Salmonellosis has been a mandatory notifiable disease in the Norwegian Surveillance System for Communicable Diseases (MSIS) since 1975. Additionally, medical microbiological laboratories are required to send all human *Salmonella* isolates to the National Reference Laboratory (NRL) for Enteropathogenic Bacteria at the Norwegian Institute of Public Health (NIPH) for identification and molecular characterization by whole genome sequencing (WGS) [4]. All *Salmonella* isolates detected in samples from animals, food or feed are routinely sent to the NRL for *Salmonella* at the Norwegian Veterinary Institute (NVI) for serotyping and WGS.

*Salmonella* Typhimurium is the second most common serovar detected in faecal samples, accounting for approximately 10–20% of all reported *Salmonella* infections in Norway. On average, approximately 50% of infections caused by *S.* Typhimurium are domestically acquired [5]. *S*. Typhimurium is one of few *Salmonella* serovars with a known reservoir among wild animals in Norway, especially passerine birds and hedgehogs [6, 7]. Animal contact and exposure to passerines or hedgehogs have previously been suggested as the primary source of infection in outbreaks of *S.* Typhimurium in humans in Norway [6, 8].

In this report we describe our results from the investigation of a national outbreak of *S.* Typhimurium sequence type 19 linked to contact with cats and passerine birds.

### Outbreak detection

On 28 June 2024, the NRL at NIPH identified a cluster of seven cases with *S.* Typhimurium sequence type (ST) 19 and cluster type (CT) 21092. The cases were geographically located in four different counties across Norway, and all were domestically acquired. The faecal samples from the cases had been collected between 6 March 2024 and 10 June 2024. A national outbreak investigation was initiated by the NIPH in collaboration with the Norwegian Food Safety Authority (NFSA), the NVI, and local health authorities in affected municipalities to identify the source of the outbreak and to prevent further cases.

## Materials and methods

### Case definition and case finding

Information about the cases, including name, sex, age and place of residence was collected from the NRL database. In addition, clinical information including onset of acute illness, symptoms, hospitalisation and place of infection was collected from MSIS.

We defined a case as:

- **Confirmed**: A person living in Norway with a laboratory-confirmed infection with *S*. Typhimurium ST19 and CT21092 and single linkage clustering with ≤ 5 allelic differences (AD) following WGS, with symptom onset or faecal sampling date after 1 March 2024.
- **Probable**: A person living in Norway with a laboratory-confirmed infection with *S*. Typhimurium and multiple locus variable-number tandem repeats analysis (MLVA) type 2-14-3-NA-0212, with symptom onset or faecal sampling date after 1 March 2024.

### Epidemiological investigation

The local food safety authorities conducted interviews with the initial eight cases using a standardized trawling questionnaire for salmonellosis. This group included the first seven cases prompting the outbreak investigation, as well as one additional case identified shortly afterward. The questionnaire included questions about symptoms, date of symptom onset, hospitalization, domestic and international travel, food consumption seven days prior to symptom onset, visit to restaurants or other places of food consumption, participation in public or private events and animal contact.

Based on results from the trawling questionnaire, a targeted interview focusing on animal contact prior to onset of symptoms was conducted for the three subsequent cases in the outbreak investigation.

The NIPH summarized and analysed the interview data. Hypotheses on possible sources of infection were established based on epidemiological data, and the information collected through the trawling questionnaire.

### Microbiological investigation

#### Human samples

All human *Salmonella* isolates received at NRL at NIPH were agglutinated with O:4, H: i and H:2 anti-serum using standard methods [9] and positive isolates were typed with MLVA for rapid identification of probable cases [10]. In parallel, WGS was performed on all *Salmonella* isolates using paired-end sequencing on the NextSeq 550 (Illumina, Inc., San Diego, US) platform aiming for a coverage of > 50x. Sequences were assembled using SPAdes v3.13.0. Species and serotype were determined using an in-house pipeline as previously described [11]. Additionally, raw sequence reads were imported to Ridom SeqSphere+ v10.0.1, to determine ST and CT as previously described [12]. Core-genome multilocus sequence typing (cgMLST) was conducted using the EnteroBase *Salmonella enterica* scheme v2 (3002 loci) [13]. Also, in SeqSphere+ serovar was assigned by *S*. enterica SISTR Geno-Serotyping v. 1.1 and antimicrobial resistance markers by NCBI AMRFinderPlus software version 3.11. The allelic profiles of the isolates were visualized as a minimum spanning tree (MST) using the parameter ‘pairwise ignoring missing values’. A cluster was defined as five or more isolates from non-travel-related cases with ≤ 5 AD based on cgMLST using single linkage clustering. Sequences from *Salmonella* detected in animal samples were compared with human isolates in the national genome database, including sequences from all human *Salmonella* cases from 2018 onwards. All sequences have been submitted to the European Nucleotide Archive (ENA) and are available through BioProject PRJEB65328 (ERR15109238-ERR15109250; including eleven human sequences and two animal sequences).

#### Animal samples

Faecal samples were analysed using VIDAS^®^ UP Salmonella (SPT). The isolates were serotyped according to ISO 6579-3:2014, which is done for all isolates submitted to the NRL *Salmonella* at NVI. During the current outbreak investigation, *S.* Typhimurium isolated from animal samples linked to the outbreak were submitted to NRL at NIPH for MLVA and WGS analysis.

### International notification

On 1 July, an event including the genomic sequence of the outbreak strain was posted in the European Surveillance Portal for Infectious Diseases (EpiPulse) to inquire if other countries had detected cases with the outbreak strain.

## Results

### Epidemiological investigation

#### Descriptive epidemiology

**Fig. 1:**
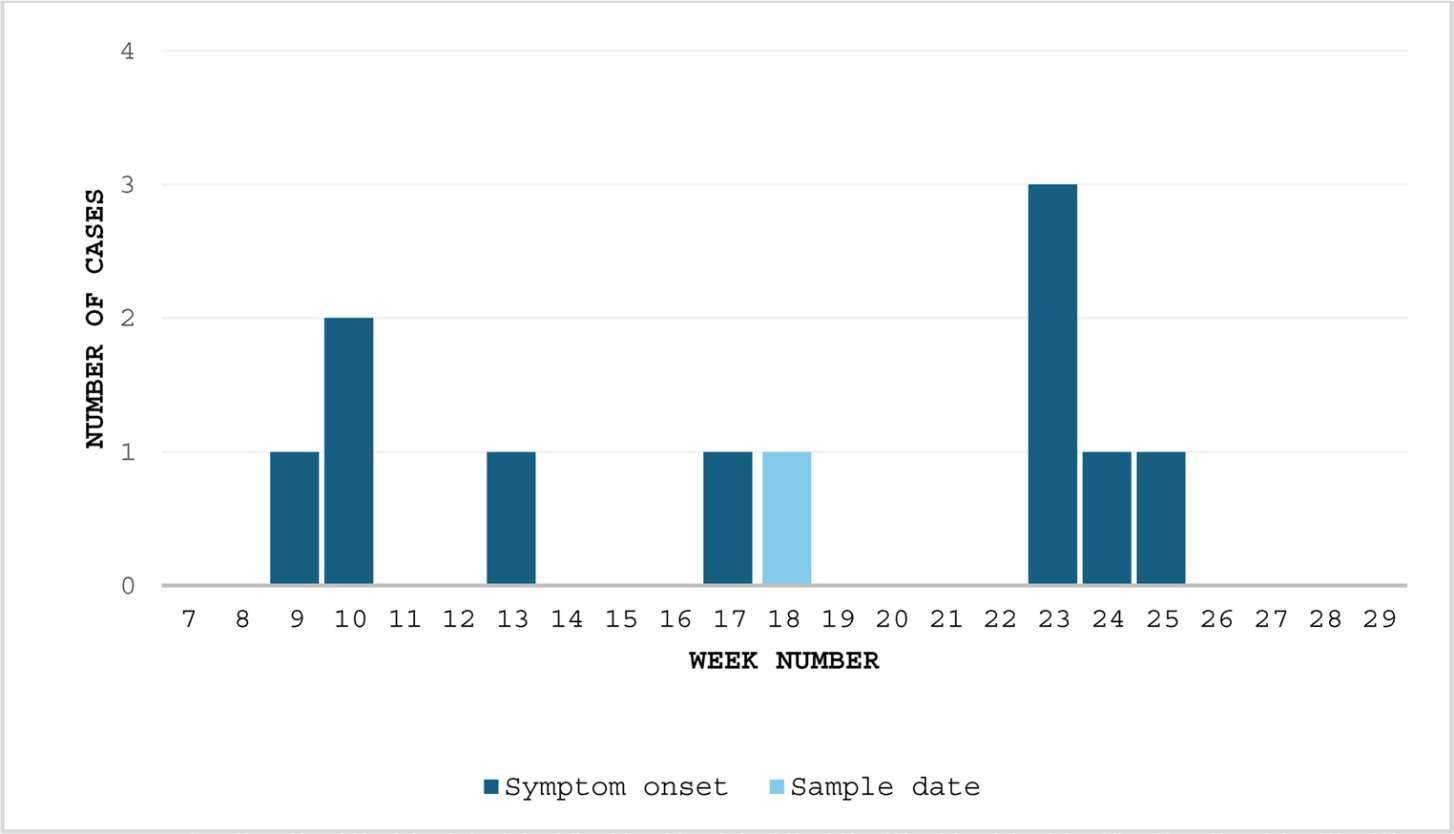
Epidemic curve (cases per week based on symptom onset, *n* = 11) in the outbreak of *Salmonella* Typhimurium ST19, CT21092, Norway, 2024. Light blue = date of symptom onset unknown, faecal sampling date used

As of 1 August 2024, eleven human cases were identified as part of the outbreak. Date of symptom onset was available for ten cases and was between 3 March and 23 June 2024 (Fig. 1). Faecal sampling dates of the cases were between 6 March and 11 July 2024.

**Fig. 2:**
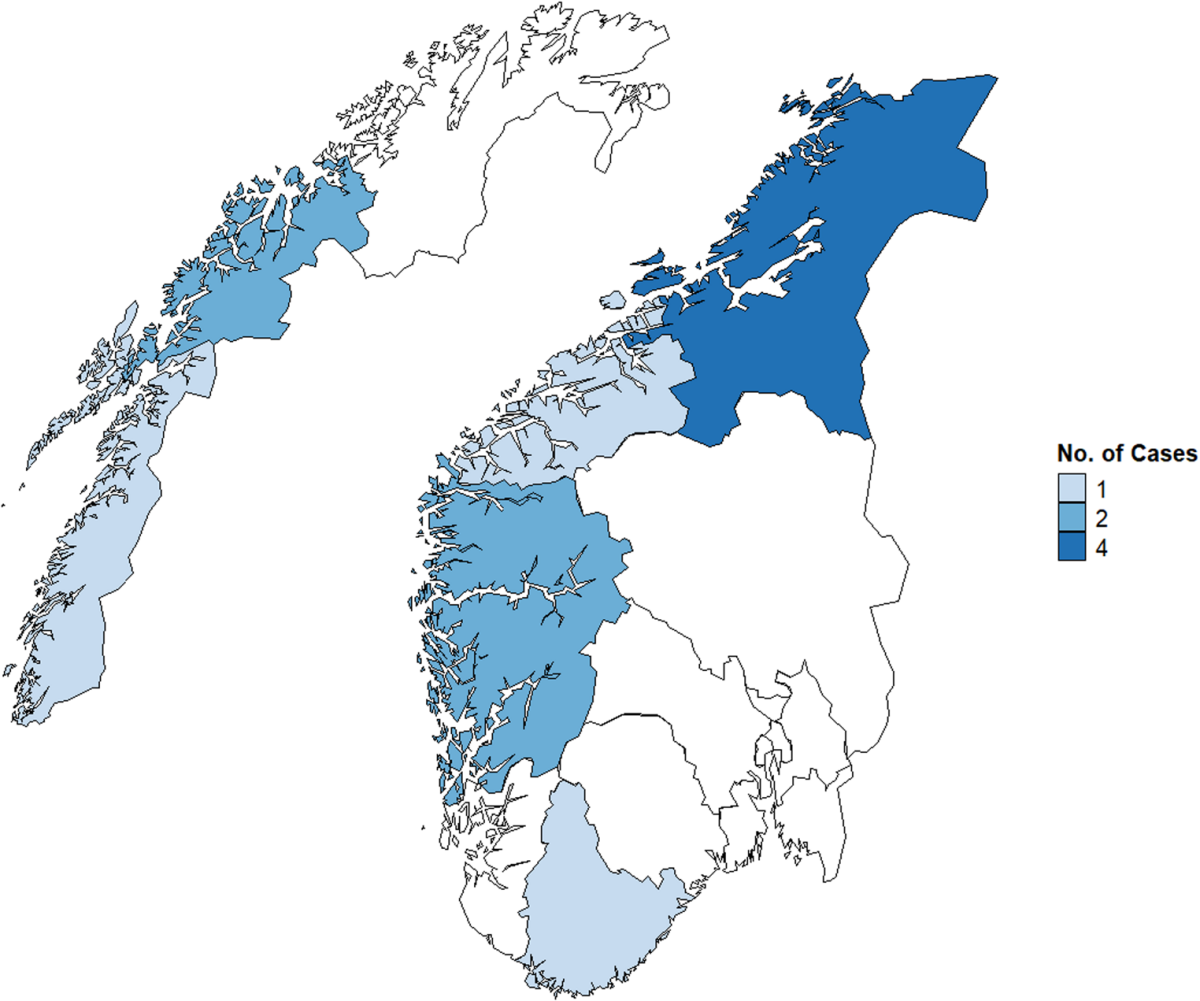
Geographical distribution of cases (*n* = 11) during the outbreak of *S.* Typhimurium ST19, CT21092, Norway, 2024

Eight cases (73%) were male and three (27%) were female. The median age of cases was 3 years (range 0–69 years). Two of the cases were siblings. Four (36%) cases were hospitalized due to their acute illness. The cases were resident in six different counties across Norway (Fig. 2). No cases reported travel abroad during the last week before onset of symptoms. Two cases reported travel within Norway, but to different parts of the country.

#### Interview of cases

**Trawling questionnaires (** ***n*** **= 8)**

Eight of eleven cases were interviewed with a trawling questionnaire (Table 1). The most commonly reported symptoms were diarrhoea (*n* = 8, 73%), fever (*n* = 5, 45%) and stomach pain (*n* = 4, 35%).

**Table 1:** The most frequently reported exposures for cases interviewed with trawling questionnaire (*n* = 8) in the outbreak of *S.* Typhimurium ST19, CT21092, Norway, 2024

Exposure	Number of cases (%)
Animal contact	8 (100%)
- Cat	5 (62.5%)
- Dog	3 (37.5%)
Sausages	5 (62.5%)
Bell peppers	5 (62.5%)
Poultry	4 (50%)
Minced meat, hamburgers	4 (50%)
Pizza with meat, minced meat or sausages	4 (50%)
Taco, fajitas, burritos	4 (50%)
Cooked ham	4 (50%)
Eggs	4 (50%)
Cucumber	4 (50%)
Carrot	4 (50%)
Chocolate	4 (50%)

In the trawling questionnaire, all eight cases reported animal contact during the last week before onset of symptoms, with five (62.5%) reporting contact with cats and three (37.5%) reporting contact with dogs (Table 1). One of the cases who reported contact with dogs also reported contact with passerines.

The most frequently reported food items were sausages and bell peppers, both reported by five cases (62.5%). All other food items were reported by ≤ 4 cases (Table 1). No common product names were reported, and food purchases had been made from different chains of grocery stores. Two of the cases were < 1 year and had primarily consumed jarred baby food. Four of eight cases reported visits to restaurants during the week prior to symptom onset, but no common restaurants were identified.

**Targeted questionnaire (** ***n*** **= 3)**

Since all cases interviewed with a trawling questionnaire reported animal exposure, the remaining three cases were subsequently interviewed with a targeted questionnaire regarding animal contact prior to onset of symptoms. All three cases reported animal exposure, with two cases reporting contact with cats and one case reporting contact with dogs.

In summary, eight of eleven cases (73%) reported contact with cats or passerine birds during the last week before symptom onset (Online resource). Seven cases had cats in their household, while one case reported contact with a diseased bird in kindergarten. The cases who did not report contact with cats or passerines, all reported contact with dogs. In addition, one of them, a small child, had been crawling on the ground outdoor (e.g. in the garden) the week prior to onset of symptoms.

### Microbiological investigation of human and animal isolates

**Fig. 3:**
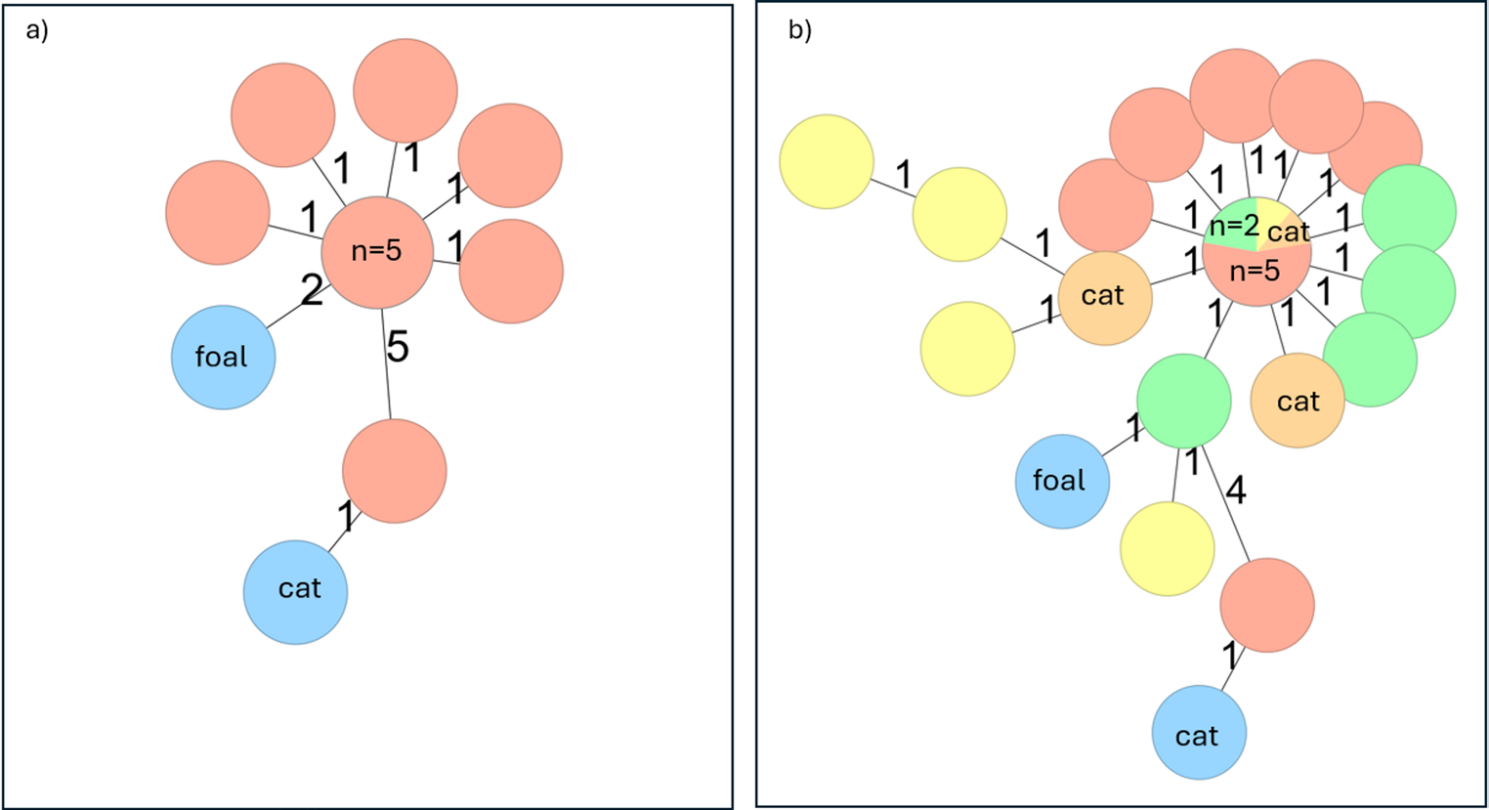
Minimum spanning tree (MST)^1^ based on core-genome MLST (cgMLST) analysis – outbreak of *Salmonella* Typhimurium ST19, CT 21,092 in Norway, 2024, (**a**) Norwegian isolates, (**b**) Norwegian, Swedish and Finnish isolates. ^1^Numbers on branches in the minimum spanning tree indicate the number of allelic differences between isolates. The number of isolates is indicated in each circle. Isolates from Norwegian human cases (*n* = 11) are in red, isolates from a cat belonging to the household of one of the cases and from a foal (*n* = 2) are in blue, isolates from Swedish cases (*n* = 6) are in green, isolates from Finnish human cases (*n* = 5) are in yellow colour and isolates from Finnish cats (*n* = 3) are in orange

*S*. Typhimurium ST19, CT21092, was detected in faecal samples from all the human cases (*n* = 11), and all the sequences were clustering with ≤ 5 AD (Fig. 3). The MLVA profile of the outbreak strain (2-14-3-NA-0212) was identical to profiles previously seen in isolates from both humans and passerine birds in Norway (2011–2017) [14]. However, the outbreak sequence had not previously been described in the national WGS database at NIPH, which includes human *Salmonella* isolates from 2018 onwards. The outbreak strain carried no antimicrobial resistance markers.

*S*. Typhimurium was isolated from a faecal sample from a cat belonging to the household of one of the cases. The cat had exhibited gastrointestinal symptoms in the days prior to illness in the owner. *S*. Typhimurium was also isolated from a faecal sample of a foal, which had no epidemiological link to the human cases. The foal had displayed symptoms of fever and diarrhoea. Additionally, *S*. Typhimurium was isolated from an environmental sample on the same farm. Both the cat and foal *S*. Typhimurium isolates had the MLVA outbreak profile and were confirmed as part of the outbreak by WGS (Fig. 3).

### International investigation

In EpiPulse, Sweden reported six human cases with isolates closely related to the outbreak strain in Norway (ENA accession no. ERR15301550-ERR15301555) (Fig. 3). The Swedish cases had faecal sampling dates between 25 April and 5 August 2024 and none of the cases reported travel abroad in the period before onset of symptoms. One of the cases had been hospitalized. Information regarding hospitalization was not available for the other cases. Two of the cases had a cat in their household. For the remaining four cases information regarding exposure to cats or birds was not available (Table 2 and Online resource).

**Table 2:** Descriptive characteristics of Norwegian (*n* = 11), Finnish (*n* = 5) and Swedish (*n* = 6) cases linked to the outbreak of *Salmonella* typhimurium ST19, CT 21092

Country	Number of cases	Median age of cases	Age range	Sex distribution (M=male, F=female)	Number of hospitalized cases (%)	Animal exposure – number of cases
Norway	11	3 years	0–69 years	8 M, 3 F	4 (36%)	Cat – 7 Dog − 5 Birds − 1
Finland	5	32 years	1–74 years	1 M, 4 F	1 (20%)	Cat – 2^1^ Birds – 2^1^ Swine − 2
Sweden	6	68.5 years	2–88 years	3 M, 3 F	1 (17%)	Cat – 2

^1^ Three Finnish cases reported contact with cats and/or birds (cats = 1, birds = 1, cats and birds = 1)

Similarly, Finland reported five human cases infected with the outbreak strain (ENA accession no. ERR15109251-15109255) (Fig. 3). *S.* Typhimurium was isolated from faecal samples in four cases and from blood culture in one case. The sampling dates were between 23 April and 7 June 2024. One of the Finnish cases was hospitalized (Table 2). All cases reported exposure to animals, whereof one case reported contact with cats, one with birds and one with both cats and birds. The last two cases reported exposure to swine (Table 2 and Online resource). Additionally, the outbreak strain was detected in faecal samples from three cats in Finland, with sampling dates corresponding with the time of illness for the human cases (ERR15109256-15109258) (Fig. 3).

### Control measures

To inform the public, information regarding the outbreak was disseminated through national media to raise public awareness about the importance of adhering to prevention measures, particularly hand hygiene measures, following contact with cats and passerine birds.

## Discussion

We report an outbreak of *S.* Typhimurium in Norway associated with exposure to cats and passerine birds in the summer of 2024. All cases had animal exposure prior to their acute illness and more than 70% of the cases had been exposed to cats or passerines during the week prior to onset of symptoms. The MLVA-profile had previously been seen in isolates from humans and passerine birds in Norway, and the outbreak strain was detected in a faecal sample from a cat owned by one of the cases, who had displayed symptoms compatible with salmonellosis prior to the onset of symptoms in the owner. This supported the hypothesis that exposure to cats and passerines was the source of this outbreak. Additionally, the outbreak strain was found in a faecal sample from a foal in another part of Norway, with no epidemiological link to the human cases. This finding, together with the findings in humans and cats in Sweden and Finland, supported the assumption that the outbreak strain had a broad environmental presence and could have spread via passerine birds.

Passerine birds are a known reservoir of *S.* Typhimurium in Norway [7], contributing to the widespread geographical distribution of *Salmonella* strains in the environment. Cats can become infected when they catch or eat passerine birds [15, 16]. Humans can become infected through direct contact with infected animals, or indirectly through exposure to litter boxes, soil, food or water contaminated with faeces from infected cats or birds. Studies investigating reservoirs of *S.* Typhimurium in the Norwegian fauna have suggested a higher risk of infection from contaminated outdoor environments in the youngest age groups [7]. Particularly in children, passerine birds on feeding boards are a known source of *Salmonella* infection [8]. Outbreaks of *S.* Typhimurium linked to contact with passerine birds have previously been reported in many countries [15, 17–20].

Two of the Finnish cases reported no contact with passerine birds or cats; however, they did report contact with swine prior to onset of symptoms. The potential for bidirectional transmission of *Salmonella* between wild birds, including passerines, and pigs has been suggested in previous studies [21, 22].

In May 2024, NVI reported an increase in *Salmonella* infections in cats. *S.* Typhimurium was detected in samples from 126 cats from different parts of Norway during late winter and spring 2024 [23]. The increase in *Salmonella* infections in cats was suggested to be related to salmonellosis in passerine birds, which in general shows a seasonal trend with a peak between February and April [24]. The winter of 2024 experienced heavy snowfall in Norway, which may explain a somewhat later peak in the incidence of *Salmonella* infections among cats this year. In winter, access to feed is often limited for passerines, and undernourishment increases their vulnerability to infections. Crowding of birds in relation to bird feeders increase the risk of transmission between individuals [24]. Passerines with salmonellosis are often weakened and become an easier prey for cats, thus increasing the risk of transmission of *Salmonella* from birds to cats.

In Norway, the cases were primarily small children and from various regions of the country, which are characteristics previously reported in outbreaks linked to bird exposure in Norway [7, 8]. However, the threshold for seeking health care and testing of children may be lower than for other age groups, thus influencing the demographic profile of cases in the outbreak. Looking at the cases from all three countries combined there was a broad age distribution, from children under the age of 1 year to elderly people, and a relatively even sex distribution. However, the total number of cases is limited, making it difficult to draw conclusions. In general, there is a predominance of cases in the youngest age groups when examining clusters related to cats and passerine birds in all three countries over the last decade.

The notification rates of human *Salmonella* infections in the Nordic countries are lower than the EU/EEA average [1, 25, 26]. Norway, Finland and Sweden are all countries with specific guarantees related to *Salmonella* in food-producing animals and thus have low presence of *Salmonella* in domestically produced food products. This may contribute to raised awareness and lower threshold for initiating outbreak investigations when domestic clusters are detected [27]. 

All three countries had cases which occurred during the same period. The presence of the same outbreak strain in all three countries underscores the importance of cross-border collaboration to detect outbreaks involving several countries. Multidisciplinary collaboration between public health institutes, veterinary institutes and food safety authorities, both domestically and internationally, is essential for a one health approach, and it can contribute to strengthen hypotheses or identifying the source of infection during outbreaks. Sharing of WGS data is of crucial importance for detection of international clusters.

In addition to the eleven cases identified in this outbreak, the outbreak strain has been identified in ten human cases in Norway and one case in Sweden in 2025. Analysis of data from the national *Salmonella* genomic database, show different clusters of *S.* Typhimurium ST19 strains spanning over several years, where passerine birds and cats are the suspected source, as the strains are closely related to animal isolates detected by the NVI. Cases belonging to the clusters are occurring throughout the year, but with a higher frequency in the 2nd quarter of the year. Also, we observe a higher proportion of females and young children (0–9 years) among the cases. These clusters highlight exposure to passerine birds and cats as a potential source of infection for human salmonellosis in Norway.

The major limitation of this outbreak investigation was the small number of reported cases, allowing only descriptive analysis and comparison of human and animal *Salmonella* strains. Furthermore, passive surveillance of salmonellosis only captures cases that seek health care and are tested, thus overrepresenting those with more pronounced illness and symptoms. Although the outbreak strain was detected in five animal samples (cats and a foal) in Norway and Finland, we have no evidence of the presence of the outbreak strain in passerine birds as no passerine samples were tested or typed for *Salmonella* in Norway in 2024.

## Conclusions

This outbreak investigation highlights the importance of communication and collaboration between public health and veterinary sectors both nationally and internationally. Identifying zoonotic outbreak sources is essential to enhance our understanding of reservoirs and circulating *Salmonella* strains in wild animals, both domestically and across borders. Our conclusion of passerines as the basis of the infections in this outbreak was based on exposure data in the present and previous outbreaks of *S*. Typhimurium and concurrent microbiological findings in three Nordic countries. The outbreak underscores the need for increased public awareness regarding passerine birds and cats as potential sources of *Salmonella* infection. Information on the importance of proper hand hygiene measures after contact with cats and birds, including bird feeders and feeding boards, should be shared in timely and efficient manner with the public.

## Supplementary Information

Below is the link to the electronic supplementary material.

Supplementary Material 1 (DOCX 32.0 KB)

## Data Availability

No datasets were generated or analysed during the current study.
