# Supplementary material for: Outbreak of Salmonella Typhimurium ST19 linked to passerine birds and cats in Norway, March to July 2024
Source: Eur J Clin Microbiol Infect Dis. 2026 Feb 5;45(5):1401–8. doi: 10.1007/s10096-026-05421-8 (PMC13222265; doi:10.1007/s10096-026-05421-8)
Supplement: Supplementary file 1 — Supplementary Material 1 (DOCX 32.0 KB) [file 10096_2026_5421_MOESM1_ESM.docx]

***Online Resource: Supplementary results***

**Article title**: **Outbreak of *Salmonella* Typhimurium ST19 linked to passerine birds and cats in Norway, March to July 2024**

**Authors:** Hilde Marie Lund (0009-0009-3290-4470) ^1^, Lin T. Brandal (0000-0003-2514-7933) ^1^, Liz Ertzeid Ødeskaug (0009-0002-5336-9015) ^1^, Heidi Lange ^1^, Polina Katsiouleri ^2^, Gro Johannessen (0000-0001-8203-2361) ^3^, Bjarne Bergsjø (0009-0003-3318-5793) ^3^, Åsne Sangolt ^4^, Rikard Dryselius ^5^, Nadja Karamehmedovic ^6^, Henry Kuronen (0009-0006-3926-9762) ^7^, Anni Vainio (0009-0009-3276-9192)^8^, Ruska Rimhanen-Finne (0000-0003-2867-7921) ^8^, Umaer Naseer (000-002-1639-8397) ^1^.

**Affiliations:**

^1^ Department of Infection Control and Preparedness, Norwegian Institute of Public Health, Oslo, Norway

^2^ Department of Bacteriology, Norwegian Institute of Public Health, Oslo, Norway

^3^ Norwegian Veterinary Institute, Ås, Norway

^4^ Regulations and Control Department, Norwegian Food Safety Authority, Norway

^5^ Department of Communicable Disease Control and Health Protection, Public Health Agency of Sweden, Solna, Sweden

^6^ Department of Microbiology, Public Health Agency of Sweden, Solna, Sweden

^7^ Laboratory and Research Division, Finnish Food Authority, Kuopio, Finland

^8^ Department of Public Health, National Institute for Health and Welfare (THL), Helsinki, Finland

**Corresponding author**: Hilde Marie Lund ([hildemarie.lund@fhi.no).](mailto:hildemarie.lund@fhi.no)

**Journal name:** European Journal of Clinical Microbiology & Infectious Diseases

| **Case ID** | **Age** | **Sex** | **County** | **Hospitalized** | **Travel history** | **Animal contact** | **ENA accession no.** |
| --- | --- | --- | --- | --- | --- | --- | --- |
| **NO-1** | 6 | F | Trøndelag | Yes | No | Cat | ERR15109238 |
| **NO-2** | 0 | M | Trøndelag | Yes | No | Cat | ERR15109239 |
| **NO-3** | 40 | M | Agder | No | No | Cat | ERR15109240 |
| **NO-4** | 39 | M | Trøndelag | Yes | No | Cat | ERR15109241 |
| **NO-5** | 3 | F | Trøndelag | No | No | Cat, dog | ERR15109242 |
| **NO-6** | 3 | F | Troms | Yes | No | Cat | ERR15109243 |
| **NO-7** | 69 | M | Møre og Romsdal | No | No | Dog | ERR15109244 |
| **NO-8** | 0 | M | Vestland | No | No | Dog | ERR15109245 |
| **NO-9** | 2 | M | Nordland | No | No | Dog | ERR15109246 |
| **NO-10** | 4 | M | Vestland | No | No | No | ERR15109247 |
| **NO-11** | 1 | M | Troms | No | No | Cat | ERR15109248 |
| **FI-1** | 2 | F | Ostrobothnia | no | no | Cat | ERR15109251 |
| **FI-2** | 74 | F | Kainuu | yes | no | Bird | ERR15109252 |
| **FI-3** | 1 | F | Southwest Finland | no | no | Swine | ERR15109253 |
| **FI-4** | 55 | M | South Ostrobothnia | no | no | Cat, bird | ERR15109254 |
| **FI-5** | 32 | F | Southwest Finland | no | no | Swine | ERR15109255 |
| **SE-1** | 88 | M | Västernorrland | Unknown | No | Unknown | ERR15301550 |
| **SE-2** | 80 | M | Dalarna | Unknown | No | Cat | ERR15301551 |
| **SE-3** | 48 | F | Norrbotten | Yes | No | Unknown | ERR15301552 |
| **SE-4** | 65 | F | Jämtland | Unknown | No | Unknown | ERR15301553 |
| **SE-5** | 2 | F | Värmland | Unknown | No | Cat, bird bath | ERR15301554 |
| **SE-6** | 72 | M | Stockholm | Unknown | No | Unknown | ERR15301555 |

**Table S1.** Descriptive characteristics of Norwegian (n=11), Finnish (n=5) and Swedish (n=6) cases linked to the outbreak of *Salmonella* Typhimurium ST19, CT 21092.
